# *Escherichia coli* ST471 Producing VIM-4 Metallo-β-Lactamase in Colombia

**DOI:** 10.1089/mdr.2021.0031

**Published:** 2022-03-04

**Authors:** Ana Mercedes Rada, Adriana Correa, Eliana Restrepo, Cesar Capataz

**Affiliations:** ^1^ Department of Microbiology, Bacteria and Cáncer Group, University of Antioquia, Medellín, Colombia.; ^2^ Facultad de Ciencias de la Salud, Biociencias Group, Institución Universitaria Colegio Mayor de Antioquia, Medellín, Colombia.; ^3^ Facultad de Ciencias Básicas, Universidad Santiago de Cali, Cali, Colombia.; ^4^ Clínica Imbanaco, Cali, Colombia.; ^5^ Fundación Clínica del Norte, Bello, Colombia.

**Keywords:** *Escherichia coli*, metallo-β-lactamase, *bla*_VIM-4_, colonization

## Abstract

An *Escherichia coli* isolate sequence-type 471 (ST471) producing Verona integron-encoded metallo-β-lactamases (VIM)-4 was recovered from a rectal swab in a patient without travel records with osteomyelitis in Colombia. The isolate carried a class 1 integron-borne *bla*_VIM-4_ gene with a 170-bp duplication in the 3′ end of the gene, preceded by an *aac(6*′)-Ib gene. The genetic environment of *bla*_VIM-4_, *bla*_CMY-2_, and *sul2* genes showed similarities to the backbone of pKKp4, an IncA/C-type plasmid from a *Klebsiella pneumoniae* strain carrying *bla*_VIM-4_ recovered in Kuwait. This is the first report of *bla*_VIM-4_ in *Enterobacterales* in South America. Our results suggest that *bla*_VIM-4_ gene was found on an IncA/C-type plasmid that could play a role in the spread of VIM-4 carbapenemase in Colombia.

The metallo-β-lactamases (MBLs) are Zn(II)-dependent enzymes that catalyze the hydrolysis of virtually all β-lactam drugs, with the exception of monobactams (*e.g.,* aztreonam). The MBLs are not inhibited by the clinically available β-lactamase inhibitors such as sulbactam, clavulanic acid, tazobactam, and avibactam, but are inhibited by chelating agents such as ethylenediaminetetraacetic acid (EDTA).^1^ These enzymes have been of clinical interest due to their broad spectra of *in vitro* resistance. They are plasmid encoded, facilitating their rapid dissemination by horizontal transfer among Gram-negative bacteria, and their appearance together with other resistance mechanisms has narrowed down treatment options for infections caused by these bacteria.^2,3^

MBLs belong to the Ambler molecular classification class B, and are divided into three subclasses B1, B2, and B3, of which the B1 subclass enzymes have emerged as the most clinically relevant. Currently, the Verona integron-encoded MBLs (VIM) constitute one of the largest groups of this subclass.^1^ VIM-1 was the first variant described in Italy (Verona) in 1997,^4^ followed by VIM-2 in France, in a carbapenem-resistant *Pseudomonas aeruginosa* clinical isolate,^5^ which is the most common MBL reported worldwide.^6^

However, other variants, such as VIM-4, have been frequently reported in strains of the *Enterobacterales* in European, African, and Asian countries.^7–10^ In Latin America, VIM-2 is the allelic variant most commonly reported in Chile, Venezuela, Brazil, Peru, Uruguay, and Argentina in *P. aeruginosa* isolates.^11^ In Colombia, VIM was first detected in 2004, identified in an outbreak of multidrug-resistant *P*. *aeruginosa* harboring VIM-8.^12^

Later, VIM-2 was detected in *P. aeruginosa*, and VIM-24 in *Klebsiella pneumoniae* in several cities in the country.^13–15^ The dissemination of MBLs in Colombia, particularly VIM-2, has been attributed to the high-risk clone of *P. aeruginosa* of sequence-type 111 (ST111).^16^ Here, we report the first detection of VIM-4 in *Enterobacterales* in South America, in *Escherichia coli* isolated from rectal swabbing of a patient from a third-level hospital in Antioquia, Colombia.

The male patient from a Colombian rural area had a history of closed fracture of the left tibia, with subsequent osteomyelitis associated with osteosynthesis material and treated initially with ciprofloxacin (initial infectious agent is unknown). The patient was then referred to third-level clinic for orthopedic management and coverage for ulcer plastic surgery.

Given the presence of an active ulcer and ciprofloxacin exposition, rectal screening was performed to detect colonization with multidrug-resistant organisms. This study identified a carbapenem-resistant *E. coli* (strain C2-70). Contact isolation measures, including strict hand washing, were implemented during the hospital stay. This report was approved by the Institutional Review Board (IRB) of the hospital.

Bacterial identification and antimicrobial susceptibility testing performed with Vitek-2 system (bioMérieux Marcy-l'Étoile, France) in the strain isolated revealed resistance to amikacin (minimum inhibitory concentration [MIC], ≥64 mg/L), gentamicin (MIC, ≥16 mg/L), ciprofloxacin (MIC, ≥64 mg/L), ampicillin-sulbactam (MIC, ≥32 mg/L), piperacillin-tazobactam (MIC, ≥128 mg/L), cefoxitin (MIC, ≥64 mg/L), ceftriaxone (MIC, ≥64 mg/L), ceftazidime (MIC, ≥64 mg/L), cefepime (MIC, 8 mg/L), ertapenem (MIC, 2 mg/L), and imipenem (MIC, 2 mg/L). The MIC results were interpreted following the Clinical and Laboratory Standards Institute (CLSI) breakpoints.^17^

In addition, the boronic acid-based combined disk test, using imipenem, yielded a negative result, and the double-disk synergy test between meropenem and imipenem versus EDTA yielded a positive result that indicated the production of an MBL.^18^ Molecular testing by PCR confirmed that the isolate harbored *bla*_VIM_ gene.^19^

Later, genomic DNA of the strain was extracted with the GeneJET Genomic DNA Purification Kit (Thermo Fisher Scientific, Waltham, MA). DNA libraries were prepared using a NexteraXT index primer kit on the Illumina platform (Illumina, San Diego, CA). Genomic libraries were sequenced on a MiSeq sequencer to obtain 250-bp paired-end reads. The readings were cleaned and assembled by *de novo* assembly using CLC Genomics Workbench, version 8.5.

The sequence data from the isolate can be found at the BioProject no. PRJNA391501; BioSample no. SAMN07291405 in the NCBI GenBank database. The assembly was typed on the web server of the Center for Genomic Epidemiology using the multilocus sequence typing (MLST).^20^ The resistance elements, the plasmid replicon, and plasmid multilocus sequence type (pMLST) were determined using Resfinder 4.1,^21^ plasmid finder 2.1, and pMLST 2.0,^22^ respectively.

The isolate belonged to sequence-type 471 (ST471) and carried several resistance genes, including *bla*_VIM-4_, *bla*_CTX-M-15_, and *bla*_CMY-2_, which encode resistance to β-lactams; *aac(6')-Ib, aac(6')-Ib-cr, aac(6')-Ian, aadA5,* and *aac(3)-IIa*, which encode resistance to aminoglycosides; *sul1* and *sul2,* which encode resistance to sulfonamides*; dfrA17* that encodes resistance to trimethoprim; *mph(A)* that encodes resistance to macrolide; *tet(B)* that encodes resistance to tetracycline; and *catB3* that encodes resistance to chloramphenicol. To this isolate belonged three plasmid replicons, including IncA/C, IncFIA, and IncFIB. The IncA/C plasmid was determined to be ST3 at the pMLST.

A BLASTn analysis indicated that the genetic environment of *bla*_VIM-4_ gene in the *E. coli* C2-70 isolate was located on the contig 7-1. The *bla*_VIM-4_ gene with a 170-bp duplicated region in the 3′ end of the gene and preceded by an *aac(6*′)-Ib gene cassette showed 100% identity to a region containing *bla*_VIM-4_ previously characterized in *K. pneumoniae* strain KP3686 (accession no. GU181265) recovered in Hungary in 2009^23^ (Fig. 1). Furthermore, the *qacEDelta1*-*sul1*-*aac*–*tnp*A IS*6100* structure was present downstream of 170-bp duplicated region, as shown in Fig. 1.

**FIG. 1.**
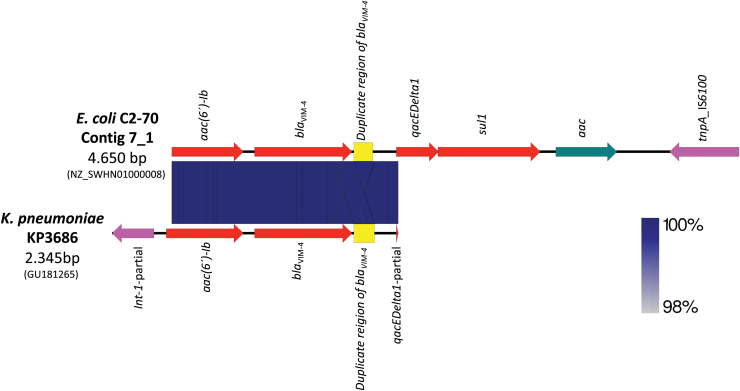
Alignment of the contig 7_1 of C2-70 isolate with the *bla*_VIM-4_ region of *Klebsiella pneumoniae* KP3686 using Easyfig. The degree of genetic similarity between the sequences is depicted by the *shaded area*, and a scale indicating the degree of similarity is at the *bottom right*. The *arrows* represent the direction of transcription. Color images are available online.

Of note, we found three contigs from *de novo* assembly of the C2-70 isolate, which showed similarities to pKKp4-VIM, an IncA/C-pST3–type plasmid of ∼165 kb (accession no. MF582638) from a *K. pneumoniae* strain carrying *bla*_VIM-4_, recovered in Kuwait, a country in Western Asia, and reported in 2017,^10^ where ∼76% of the backbone was covered. The 7_2 contig showed similarities with three resistance islands that harbored the pKKp4 (RI-1, RI-2, and RI-3) (Fig. 2).

**FIG. 2.**
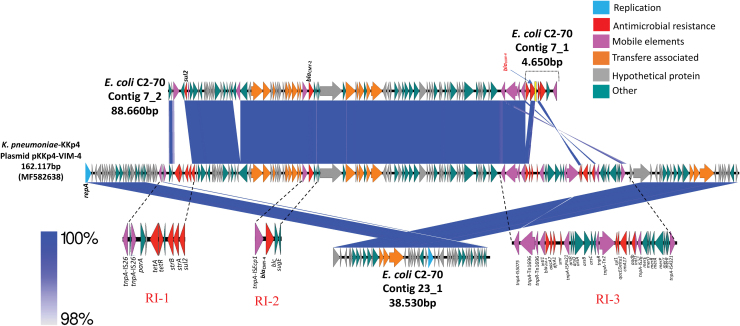
Alignment of the contig 7_2, 23_1 and 7_1 of C2-70 isolate with the plasmid pKKp4-VIM from *Klebsiella pneumoniae* KKp4 using Easyfig. The degree of genetic similarity between the sequences is depicted by the *shaded area*, and a scale indicating the degree of similarity is at the *bottom left*. The *arrows* represent the direction of transcription. Genes are *color coded* depending on functional annotations at the *bottom right*. Alignment of the plasmid pKKp4-VIM (MF582638) harboring *bla*_VIM-4_ with contigs 7_2, 23_1 and 7_1 of C2-70 with 52%, 23%, and 1% of coverage, respectively, by BLAST. The RI-1, RI-2, and RI-3 of pKKp4-VIM are also shown (*black dotted lines*). BLAST, basic local alignment search tool; VIM, Verona integron-encoded metallo-β-lactamases. Color images are available online.

In addition, we characterized the genetic location of *bla*_VIM-4_ by S1-nuclease pulsed-field gel electrophoresis (S1-PFGE) and southern hybridization using a digoxigenin (DIG) DNA-labeled *bla*_VIM_ probe (DIG-High Prime DNA labeling and detection starter kit II; Roche, Germany), following the manufacturer's instructions.^24,25^ Our results indicated that C2-70 isolate carried three plasmids of 121, 145.5, and 291 kb, the *bla*_VIM-4_ gene being harbored by the 145.5-kb plasmid (Fig. 3).

**FIG. 3.**
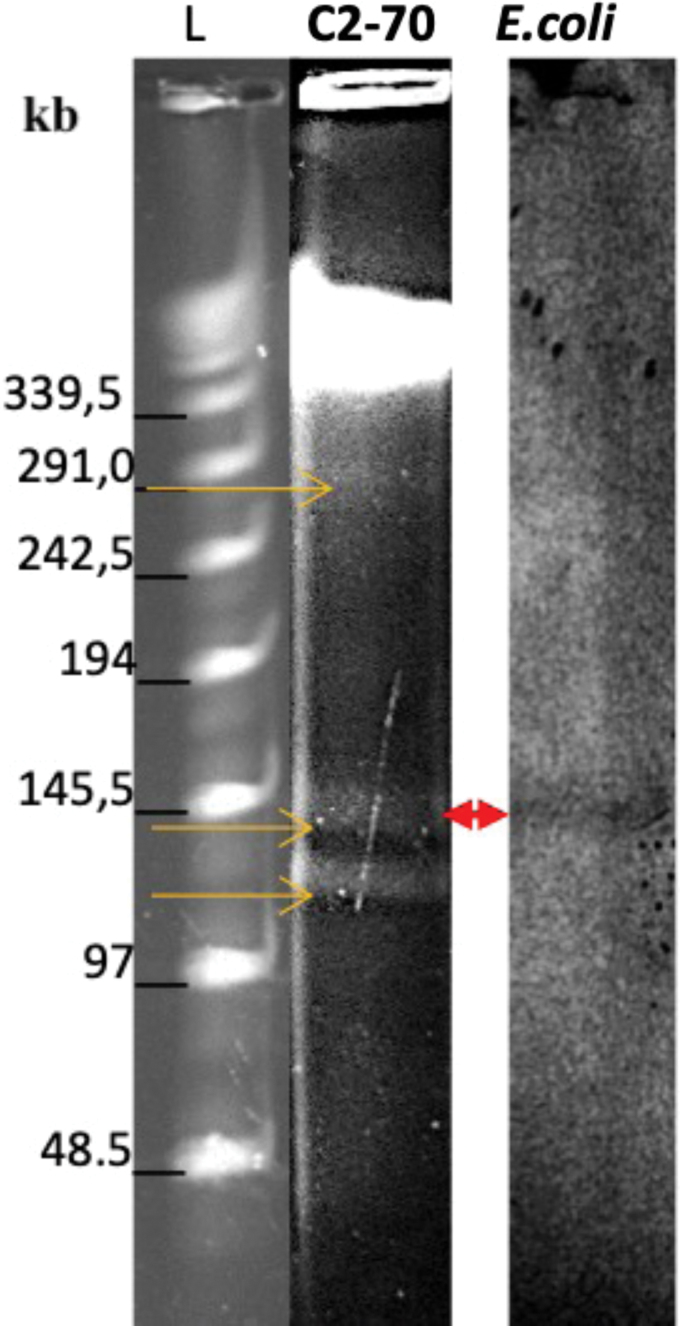
Plasmid location of *bla*_VIM-4_ on isolate C2-70 confirmed by S1-PFGE and southern hybridization with a *bla*_VIM_ probe. The plasmid bands in S1-PFGE are indicated by *yellow arrows* (*left panel*), a lambda ladder was included as reference (L). The plasmid band in which the *bla*_VIM-4_ was detectable by southern hybridization using *bla*_VIM-4_ probe is indicated by a *red triangle* (*right panel*). S1-PFGE, S1-pulsed-field gel electrophoresis. Color images are available online.

VIM-producing *Enterobacterales* have become endemic in some European countries, especially in Greece^7^; in contrast, in South America few reports exist. Until now, VIM-2, VIM-11, VIM-16, VIM-23, and VIM-24 variants have been described,^11,26^ demonstrating that in some cases the *bla*_VIM_ gene was associated with class I integron, an efficient genetic platform to incorporate MBL genes, and disseminated once it has been embedded within transposons or plasmids.^26^ To our knowledge, this is the first case report of *E. coli* harboring *bla*_VIM-4_ in South America. Of note, *bla*_VIM-4_ had not been previously reported in Colombia, an endemic country for VIM-2–producing *P. aeruginosa*.^16^

The *bla*_VIM-4_ gene is a variant of the *bla*_VIM-1_ that was first detected in *P. aeruginosa* in Greece in 2001,^27^ and later VIM-4–producing *P. aeruginosa, Aeromonas hydrophila,* and *Enterobacterales* were reported in other European countries.^28–30^ This MBL differs from VIM-1 by only one amino acid substitution (S228R) and from VIM-2 due to a different position of the flapping loop and two substitutions in loop 2, VIM-4 being more active than VIM-1 against benzylpenicillin, cephalothin, nitrocefin, and imipenem and less active than VIM-2 against ampicillin and meropenem.^31^

Interestingly, in our case we identified a patient without travel records colonized with *E. coli* ST471, a high-risk clone associated with genes encoding carbapenemases,^32^ carrying an integron-borne *bla*_VIM-4_ gene with a 170-bp duplication in the 3′ end of the gene, identical to that previously characterized in *K. pneumoniae, Klebsiella oxytoca*, *A. hydrophila,* and *P. aeruginosa* isolates in Hungary.^23,28,29^ Three plasmid replicons were identified for this isolate, including IncA/C genotyped as pST3. BLASTn analysis showed at least a coverage of 76% of the backbone of pKKp4-VIM, an endemic IncA/C-pST3–type plasmid from Kuwait that spread among *Enterobacterales* strains of *multiple species*, carrying the *bla*_VIM-4_ gene within the In416 integron.^10^

The contig 7-2 covered ∼53% of the backbone of pKKp4 and showed similarities with three resistance islands of this plasmid. The contig was 99,95% identical to the RI-2, since containing the *bla*_CMY-2_ gene instead of *bla*_CMY-4_ gene, also it was similar to the RI-3 in the region that contained the tnpA IS*5075*-tnpA IS*1696*-tnpR IS*1696*-*intI1* and the region that contained the *sul2* gene of the RI-1 (Fig. 2).

On the contrary, the contig 23_1 covered ∼23% of the backbone of pKKp4 in a region containing genes for conjugative transfer of plasmid and the repA replication gene, as shown in Fig. 2. Nevertheless, the pKKP4 did not contain the 170-bp duplicated region in the 3′ end of the *bla*_VIM-4_ gene as well as not being preceded by *aac(6*′)-Ib gene cassette identified in the contig 7_1 of the assembly of C2-70 that overlapped with contig 7_2 containing the class 1 integron (Fig. 2), similar to In238 previously reported in one isolate of *Aeromona hydrophila* from Hungary (accession no. EU581706).^29^

Overall, our results suggest that *bla*_VIM-4_ gene was found on an IncA/C-pST3–type plasmid with respective regions for replication, conjugative transfer, and plasmid maintenance, which could play a role in the spread of VIM-4 carbapenemase in Colombia. Thus, future long read sequencing studies are required to confirm the complete sequence of this plasmid.
